# The KDET Motif in the Intracellular Domain of the Cell Adhesion Molecule L1 Interacts with Several Nuclear, Cytoplasmic, and Mitochondrial Proteins Essential for Neuronal Functions

**DOI:** 10.3390/ijms24020932

**Published:** 2023-01-04

**Authors:** Ralf Kleene, Gabriele Loers, Melitta Schachner

**Affiliations:** 1 Zentrum für Molekulare Neurobiologie, Universitätsklinikum Hamburg-Eppendorf, Martinistr. 52, 20246 Hamburg, Germany; 2 Keck Center for Collaborative Neuroscience, Department of Cell Biology and Neuroscience, Rutgers University, 604 Allison Road, Piscataway, NJ 08854, USA

**Keywords:** cell adhesion molecule L1, proteolytic processing, KDET motif, neurite outgrowth, neuronal survival

## Abstract

Abnormal functions of the cell adhesion molecule L1 are linked to several neural diseases. Proteolytic L1 fragments were reported to interact with nuclear and mitochondrial proteins to regulate events in the developing and the adult nervous system. Recently, we identified a 55 kDa L1 fragment (L1-55) that interacts with methyl CpG binding protein 2 (MeCP2) and heterochromatin protein 1 (HP1) via the KDET motif. We now show that L1-55 also interacts with histone H1.4 (HistH1e) via this motif. Moreover, we show that this motif binds to NADH dehydrogenase ubiquinone flavoprotein 2 (NDUFV2), splicing factor proline/glutamine-rich (SFPQ), the non-POU domain containing octamer-binding protein (NonO), paraspeckle component 1 (PSPC1), WD-repeat protein 5 (WDR5), heat shock cognate protein 71 kDa (Hsc70), and synaptotagmin 1 (SYT1). Furthermore, applications of HistH1e, NDUFV2, SFPQ, NonO, PSPC1, WDR5, Hsc70, or SYT1 siRNAs or a cell-penetrating KDET-carrying peptide decrease L1-dependent neurite outgrowth and the survival of cultured neurons. These findings indicate that L1’s KDET motif binds to an unexpectedly large number of molecules that are essential for nervous system-related functions, such as neurite outgrowth and neuronal survival. In summary, L1 interacts with cytoplasmic, nuclear and mitochondrial proteins to regulate development and, in adults, the formation, maintenance, and flexibility of neural functions.

## 1. Introduction

The cell adhesion molecule L1 is involved in the regulation of multiple and diverse neural functions, such as proliferation, survival, and migration of neural cells; neuritogenesis, axonal outgrowth, fasciculation, and guidance of axons, as well as myelination and synaptogenesis during nervous system development (for reviews, see [1,2,3,4]). In adulthood, L1 modulates synaptic plasticity, learning, and memory (for a review, see [4]) and contributes to regeneration after injury by promoting axonal regrowth and remyelination (for references, see [5]). In humans, mutations in L1 are associated with neurological and psychiatric disorders, such as fetal alcohol syndrome, Hirschsprung’s disease, schizophrenia, Alzheimer’s disease, and L1 syndrome, which comprises a spectrum of mild to severe congenital X chromosome-linked developmental disorders [3,6,7,8,9]. In mice, L1 deficiency can lead to severe malformations and malfunctions of the nervous system [10,11,12].

The beneficial functions of L1 in the nervous system not only depend on homo- and heterophilic interactions (for references, see [4,13]), but also on the proteolytic cleavage of L1 (for references, see, for instance, [14]). L1 consists of an extracellular N-terminal part comprising six immunoglobulin-like (Ig) domains and five fibronectin type III (FNIII) domains, a transmembrane domain, and a C-terminal intracellular tail [15,16]. L1 can undergo proteolytic processing to release soluble and transmembrane fragments, which are important for the neural functions of L1 [5,17,18,19,20,21,22,23,24].

In previous studies, we have shown that stimulation of L1 signaling leads to the myelin basic protein-mediated generation of a transmembrane C-terminal fragment of 70 kDa, called L1-70, which plays important roles in neuritogenesis, neuronal migration, and neuronal survival as well as mitochondrial homeostasis [5,17,18,19,21,25]. In a more recent study, we have identified a novel C-terminal L1 fragment of 55 kDa, L1-55, which interacts with MeCP2 and the HP1 isoforms α, β, and γ, and is also involved in L1-dependent functions, such as neurite outgrowth and neuronal migration [26,27]. L1-55 binds to the HP1 isoforms α, β, and γ, as well as to MeCP2 via a KDET motif in its intracellular domain [26,27]. Besides MeCP2 and HP1, we had identified NDUFV2 as a mitochondrial binding partner [17] and, by affinity chromatography using the intracellular domain, as nuclear binding partners, SFPQ (also known as polypyrimidine tract binding associated-splicing factor or PSF), NonO (also known as 54 kDa nuclear RNA- and DNA-binding protein or p54nrb), PSPC1, estrogen receptors α (ERα) and β (ERβ), peroxisome proliferator-activated receptor γ (PPARγ), and retinoid X receptor β (RXRβ) [17,18,28] (Table 1). Here, we investigated whether these and other potential L1 binding partners [18,28], such as DNA topoisomerase I (TOP1), WDR5, HistH1e, nucleoporin 93 kDa (Nup93), Hsc70, SYT1, importin β1 (impβ1), and heterogeneous nuclear ribonucleoprotein (hnRNP) A isoforms A1, A2/B1, and A3, as well as androgen receptor (AR) and vitamin D receptor (VDR) [28] (Table 1), interact with L1-55 or other L1 fragments via the membrane-proximal KDET motif.

## 2. Results

### 2.1. Several Binding Partners Interact with L1 via the KDET Motif

**Figure 1:**
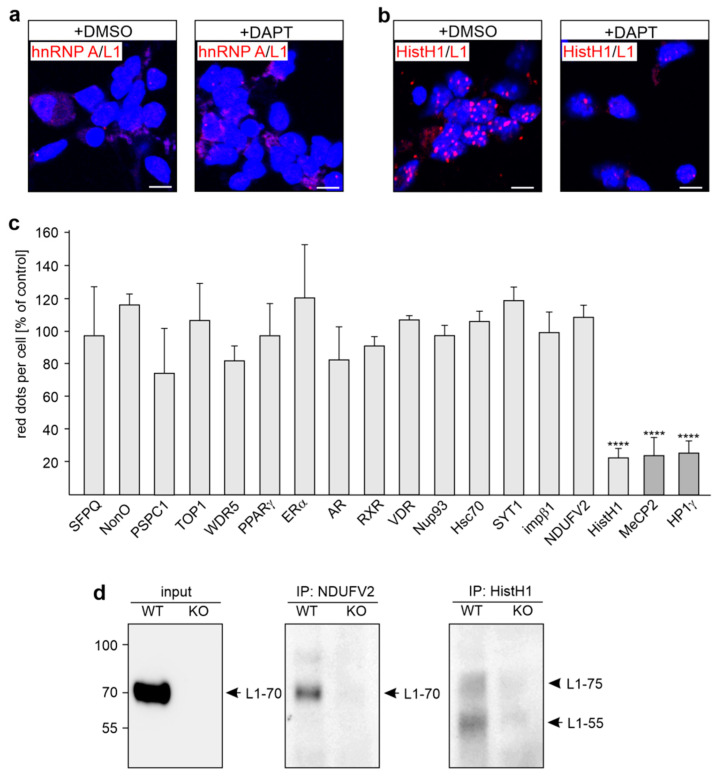
L1-55 interacts with MeCP2, HP1, and HistH1 but not with other verified or putative L1 binding partners in cultured cortical neurons. Neurons were treated with the vehicle dimethyl sulfoxide (DMSO) (+DMSO) or with the γ-secretase inhibitor DAPT and were then subjected to proximity ligation with L1 antibodies and antibodies against NDUFV2, SFPQ, NonO, PSPC1, WDR5, TOP1, hnRNP A isoforms, HistH1, Nup93, Hsc70, SYT1, impβ1, ERα, RXR, PPARγ, AR, VDR, MeCP2, or HP1γ. Nuclei are stained with DAPI (4′,6-diamidino-2-phenylindole). (**a**,**b**) Representative images of DMSO- and DAPT-treated neurons stained with mouse L1 antibody C-2 and a rabbit antibody against hnRNP A isoforms (**a**) or HistH1 (**b**) are shown. Scale bar: 10 µm. (**c**) The mean values + SD are from two independent experiments and show average numbers of red dots per cell after DAPT-treatment relative to control (values of vehicle control set to 100%) (**** *p* < 0.001; one-way ANOVA with Dunn’s multiple comparison test). Values obtained for proteins known to bind to L1-55 served as positive controls and are marked in dark gray. (**d**) Non-nuclear fractions from wild-type (WT) and L1-deficient (KO) mice were used for immunoprecipitation with mouse HistH1 or NDUFV2 antibodies immobilized to Protein G. Fractions (input) and immunoprecipitates (IP) were subjected to Western blot analysis with L1 antibody C-2. Arrows indicate L1-55 and L1-70, and the arrowhead indicates an unknown L1 band of approximately 75 kDa.

We have shown that the interaction of MeCP2 and HP1 with L1-55 is mediated by the KDET motif in the intracellular domain of L1 (L1-ICD) [26,27]. Besides MeCP2 and HP1α, -β, and -γ, we identified NDUFV2, SFPQ, NonO, PSPC1, ERα, PPARγ, RXRβ, and TOP1 as L1’s binding partners [17,18,28,29]. We now asked whether these binding partners also interact with L1-55. In parallel, we analyzed whether the putative L1 binding partners WDR5, HistH1e, Nup93, Hsc70, SYT1, impβ1, and hnRNP A isoforms [28], as well as AR and VDR, interact with L1-55. Proximity ligation, which detects interactions between proteins localized at a distance of 40 nm or less, showed that the interactions of L1-55 with its binding partners MeCP2 and HP1 are reduced by the γ-secretase inhibitor DAPT ((2S)-N-[(3,5-difluorophenyl)acetyl]-L-alanyl-2-phenyl]glycine 1,1-dimethylethyl ester) [26,27]. On the basis of these findings, cultured cortical neurons were treated with DAPT and subjected to a proximity ligation assay using L1 antibodies and antibodies against NDUFV2, SFPQ, NonO, PSPC1, WDR5, HistH1, TOP1, hnRNP A isoforms, Nup93, Hsc70, SYT1, impβ1, ERα, RXR, PPARγ, AR, and VDR to analyze whether these proteins are associated with L1-55. In parallel, proximity ligation with L1 and MeCP2 or HP1γ antibodies was performed for positive control. Using either mouse L1 and rabbit hnRNP A antibodies or rabbit L1 and mouse hnRNP A antibodies, less than 0.1 L1/hnRNP A-positive dots per cell were counted (Figure 1a), indicating that hnRNP A isoforms do not associate with L1. Higher numbers of dots (1–4 dots per cell) ascertained that NDUFV2, SFPQ, NonO, PSPC1, WDR5, TOP1, HistH1e, Nup93, Hsc70, SYT1, impβ1, ERα, RXR, PPARγ, AR, and VDR interact with L1. The numbers of HistH1/L1-positive dots were reduced in neurons treated with the γ-secretase inhibitor DAPT when compared to the numbers in vehicle-treated neurons (approximately 1 dot per cell), as seen for MeCP2 and HP1 (Figure 1b,c), indicating that HistH1e interacts with L1-55, like MeCP2 and HP1 [26,27]. The numbers of NDUFV2/L1-, SFPQ/L1-, NonO/L1-, PSPC1/L1-, WDR5/L1-, TOP1/L1-, Nup93/L1-, Hsc70/L1-, SYT1/L1-, impβ1/L1-, ERα/L1-, RXR/L1-, PPARγ/L1-, AR/L1-, and VDR/L1-positive dots were not reduced by DAPT treatment (Figure 1c), indicating that NDUFV2, SFPQ, NonO, PSPC1, ERα, RXR, PPARγ, WDR5, TOP1, Nup93, Hsc70, SYT1, impβ1, AR, and VDR are not associated with L1-55.

To verify the notion that HistH1e binds to L1-55, immunoprecipitation with HistH1 antibody and cellular subfractions from the brains of wild-type and L1-deficient mice was performed. As a negative control, we used an antibody against NDUFV2, which was shown to co-immunoprecipitate L1-70 but not L1-55 [17]. Western blot analysis indicates L1-immunopositive L1 bands of approximately 55 and 75 kDa in the HistH1 immunoprecipitate of the non-nuclear fraction from wild-type but not L1-deficient brains (Figure 1d). No bands were observed when nuclear fractions were used for immunoprecipitation. In the NDUFV2 immunoprecipitate of the non-nuclear fraction from wild-type but not L1-deficient brain, a 70 kDa L1 band was found (Figure 1d), confirming that NDUFV2 interacts with L1-70. In the wild-type input control, only L1-70 was detected, while L1-55, which is present in the brain only in low amounts [26,27], was not seen in the input (Figure 1d). The results indicate that HistH1 interacts with L1-55 and possibly with an unknown L1 fragment of 75 kDa.

**Figure 2:**
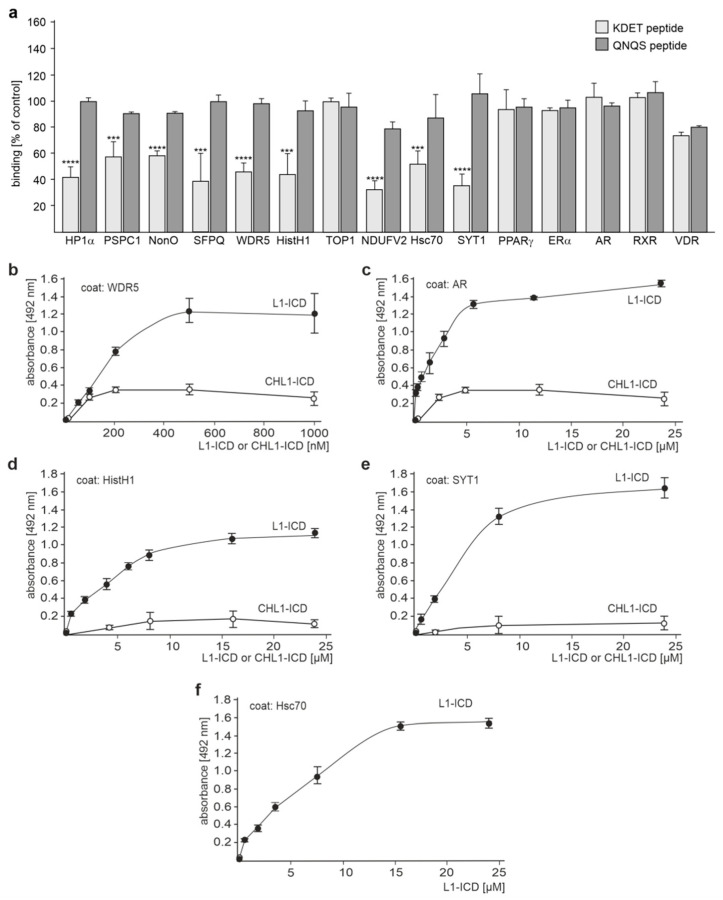
L1-ICD binds to several binding partners via its KDET motif. Recombinant L1-binding partners were substrate-coated and incubated with a constant L1-ICD concentration in the absence or presence of the KDET peptide or QNQS control peptide (**a**) or with increasing L1-ICD concentrations (**b**–**f**). Binding was determined by ELISA using mouse L1 antibody C-2 and horseradish peroxidase-conjugated secondary antibodies. The mean values ± SD from three independent experiments carried out in triplicate are shown for the binding relative to control (values in the absence of peptides set to 100%). *** *p* < 0.005, **** *p* < 0.001; one-way ANOVA with Bonferroni´s multiple comparison test.

Since recombinant SFPQ, NonO, PSPC1, and NDUFV2 bind to L1-ICD in ELISA [17,28], we performed competition ELISA with the KDET peptide to analyze whether binding of these proteins to L1-ICD is mediated by KDET. As a negative control, a peptide with a disrupted KDET motif was used. Disruption of the motif was achieved by the highly conserved substitution of K, D, E, and T by Q, N, Q, and S, respectively. We preferred this QNQS control peptide with conservative amino acid substitutions over a control peptide with a scrambled KDET sequence, because non-conservative scrambling could affect the primary structure much more than conservative substitutions, e.g., when K are exchanged by D, E, or T, when D or E is exchanged by K or T, and when T is exchanged by K, D, or E. In parallel, we tested whether L1-ICD binds to WDR5, TOP1, HistH1e, Hsc70, SYT1, ERα, RXRβ, PPARγ, AR, or VDR via the KDET motif. The interaction of L1-ICD with Nup93 and impβ1 was not analyzed, because no appropriate recombinant Nup93 and impβ1 proteins were available. The binding of L1-ICD to recombinant SFPQ, NonO, PSPC1 WDR5, NDUFV2, Hsc70, SYT1, HistH1e, and HP1α was reduced by the KDET peptide, but not by the negative control QNQS peptide, while the binding of L1 to TOP1, ERα, RXRβ, PPARγ, VDR, and AR was not affected by either peptide (Figure 2a). ELISA with substrate-coated recombinant HistH1e, WDR5, Hsc70, SYT1, or AR and different L1-ICD concentrations showed concentration-dependent binding of L1-ICD to WDR5, HistH1e, Hsc70, SYT1, and AR (Figure 2b–f), indicating that the binding of WDR5, HistH1e, Hsc70, SYT1, and AR to L1-ICD is specific and suggesting that not only AR but also other nuclear receptors, e.g., ERα, RXRβ, PPARγ, and VDR bind to L1-ICD. For negative control, CHL1-ICD did not bind to WDR5, HistH1e, SYT1, and AR (Figure 2b–eSince Hsc70 is known to bind to CHL1-ICD via the HPD motif [30], ELISA with Hsc70 and CHL1 was not performed. The results indicate that the binding of SFPQ, NonO, PSPC1 WDR5, NDUFV2, Hsc70, SYT1, and HistH1e to L1-ICD is mediated by the KDET motif.

**Figure 3:**
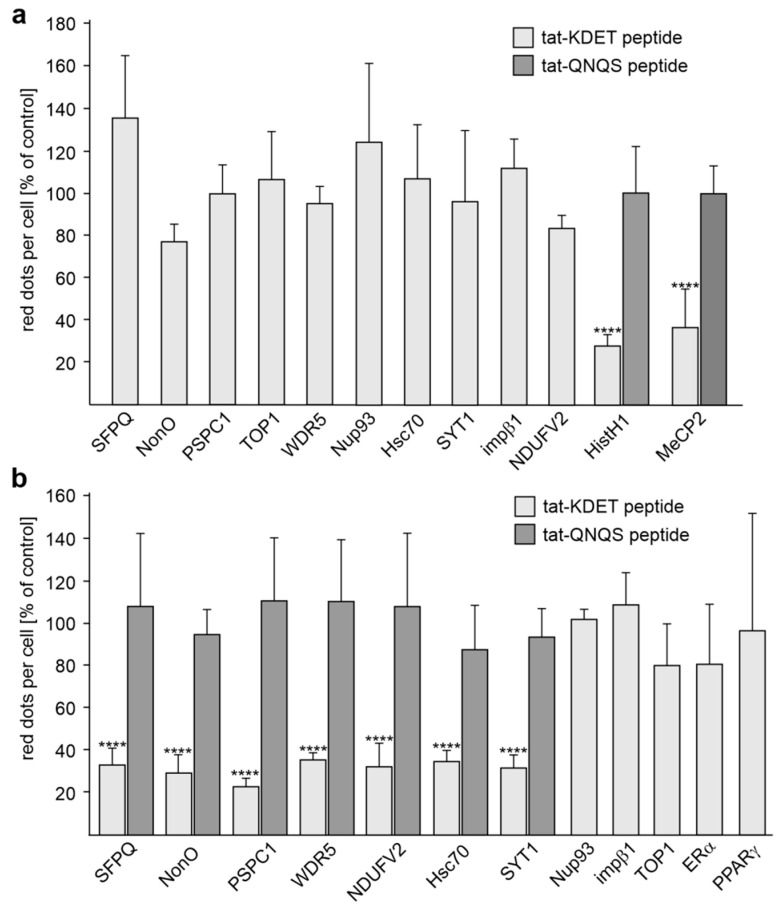
In cultured cortical neurons, L1 interacts with several binding partners via its KDET motif. Cultured cortical neurons were treated with vehicle, tat-KDET peptide, or tat-QNQS control peptide, followed by treatment without (**a**) and with (**b**) L1 antibody 557 and proximity ligation with a L1 antibody and an antibody against MeCP2, NDUFV2, SFPQ, NonO, PSPC1, WDR5, TOP1, HistH1, Nup93, Hsc70, SYT1, ERα, or PPARγ. Mean values + SD from two independent experiments are shown for the average numbers of red dots per cell relative to control (values of treatment with vehicle set to 100%) (**** *p* < 0.001; one-way ANOVA with Bonferroni´s multiple comparison test).

In the next step, we further analyzed in a cellular context whether the binding of L1 to SFPQ, NonO, PSPC1, WDR5, NDUFV2, Hsc70, SYT1, and HistH1e is mediated via the KDET motif. To this aim, cortical neurons were first incubated with or without the cell-penetrating tat-KDET peptide, then treated with or without function-triggering monoclonal L1 antibody 557, and thereafter subjected to proximity ligation with L1 antibody and an antibody against SFPQ, NonO, PSPC1, WDR5, NDUFV2, Hsc70, SYT1, or HistH1e. The tat-QNQS peptide, which carries a disrupted KDET motif, served as a negative control. As a positive control, proximity ligation with L1 and MeCP2 antibodies was performed. To test whether Nup93 and impβ1 interact with L1 via KDET, proximity ligation with L1 antibody and Nup93 or impβ1 antibody was carried out. Under basal, unstimulated conditions, the numbers of L1/HistH1- and L1/MeCP2-positive dots were reduced by the tat-KDET peptide but not by the tat-QNQS control peptide (Figure 3a). The numbers of L1/WDR5-, L1/NDUFV2-, L1/SFPQ-, L1/NonO-, L1/PSPC1-, L1/Hsc70-, L1/SYT1-, L1/Nup93, L1/TOP1-, and L1/ impβ1-positive dots were not reduced by the tat-KDET peptide (Figure 3a). After stimulation of neurons with antibody 557, the numbers of L1/NDUFV2-, L1/WDR5-, L1/SFPQ-, L1/NonO-, L1/PSPC1-, L1/Hsc70-, and L1/SYT1-positive dots were reduced by the tat-KDET peptide, but not by the tat-QNQS control peptide, while the numbers of L1/Nup93- and L1/ impβ1-positive dots were not reduced by the tat-KDET peptide (Figure 3b). Proximity ligation with a L1 antibody and an antibody against TOP1, ERα, or PPARγ, which do not interact with L1 via the KDET motif, showed that the numbers of L1/TOP1-, L1/ERα- and L1/PPARγ-positive dots in L1 antibody 557-stimulated neurons were not altered by treatment with the tat-KDET peptide (Figure 3b). These results indicate that the interactions of L1 with SFPQ, NonO, PSPC1, WDR5, NDUFV2, Hsc70, SYT1, and HistH1e are mediated by the KDET motif.

### 2.2. The KDET Motif Is Essential for L1-Dependent Neurite Outgrowth and Neuronal Survival

**Figure 4:**
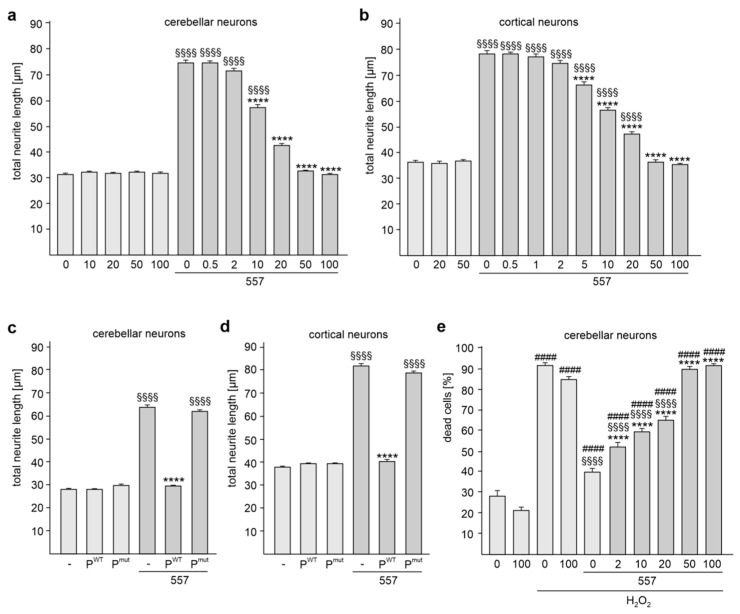
The KDET-mediated interaction of L1 with its binding partners is essential for L1-dependent neurite outgrowth and neuronal cell survival. Cerebellar (**a**,**c**) and cortical (**b**,**d**) neurons were treated with 0, 0.5, 1, 2, 5, 10, 20, 50, or 100 µg/mL tat-KDET peptide (**a**,**b**), with 0 or 50 µg/mL tat-KDET peptide (P^WT^) or with 0 or 50 µg/mL tat-QNQS peptide (P^mut^) (**c**,**d**). Neurons were then treated without or with antibody 557. Mean values + SEM from three independent experiments are shown for total neurite lengths (**** *p* < 0.0001 relative to stimulated neurons not treated with tat-KDET peptide, §§§§ *p* < 0.0001 relative to non-stimulated neurons not treated with tat-KDET peptide or tat-QNQS peptide; one-way ANOVA with Dunn’s multiple comparison test). (**e**) Cerebellar neurons were first treated with 0, 2, 10, 20, 50, or 100 µg/mL tat-KDET peptide and then treated without or with antibody 557 in the absence or presence of H_2_O_2_. Mean values + SEM from three independent experiments are shown for the relative numbers of dead cells (**** *p* < 0.0001 relative to stimulated neurons not treated with tat-KDET peptide in the presence of H_2_O_2_, §§§§ *p* < 0.0001 relative to non-stimulated neurons not treated with tat-KDET peptide in the presence of H_2_O_2_, #### *p* < 0.0001 relative to unstimulated neurons not treated with tat-KDET peptide in the absence of H_2_O_2_; one-way ANOVA with Dunn´s multiple comparison test).

Next, we investigated whether the KDET-mediated interaction of L1 with its binding partners plays a role in L1-dependent neurite outgrowth. Since treatment with L1 antibody 557 increases neurite outgrowth [19,21,22], we applied this antibody to cerebellar and cortical neurons in order to augment L1-dependent neurite outgrowth and to determine whether binding of L1 to its binding partners via the KDET motif is involved in this L1-stimulated enhancement of neurite outgrowth. Treatment with this antibody promoted neurite outgrowth in comparison to non-treated neurons (Figure 4a,b). This enhanced neurite outgrowth was reduced in cerebellar and cortical neurons by treatment with the cell-penetrating tat-KDET peptide in a concentration-dependent manner (Figure 4a,b). The peptide did not affect neurite outgrowth from non-stimulated neurons (Figure 4a,b). Treatment of cerebellar and cortical neurons with the tat-KDET peptide, but not the tat-QNQS peptide, inhibited L1-induced neurite outgrowth from cerebellar and cortical neurons (Figure 4c,d). These results indicate that L1-mediated neurite outgrowth depends on the KDET-mediated interaction of L1 with its binding partners.

Since treatment with antibody 557 also protects neurons against hydrogen peroxide-induced oxidative stress [31,32], we analyzed whether the KDET-mediated interaction of L1 and its binding partner is involved in neuronal survival. The hydrogen peroxide treatment increased cell death, which was reduced in the presence of antibody 557 (Figure 4e). The antibody 557-mediated cell survival of cerebellar neurons was reduced by the tat-KDET peptide in a concentration-dependent manner (Figure 4e). The peptide did not affect hydrogen peroxide-induced cell death in the absence of L1 antibody 557 (Figure 4e). These results indicate that L1-mediated neuronal survival also depends on the KDET-mediated interaction of L1 with its binding partners.

### 2.3. Reduction of HistH1e, NDUFV2, SFPQ, NonO, WDR5, Hsc70, or SYT1 Expression Decreases L1-Dependent Neurite Outgrowth

**Figure 5:**
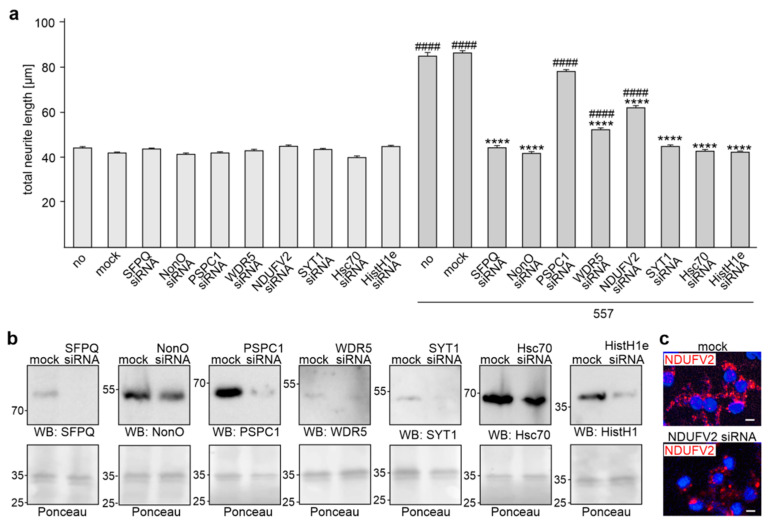
Reduction of SFPQ, NonO, WDR5, NDUFV2, SYT1, Hsc70, and HistH1e expression by siRNAs inhibits L1-dependent neurite outgrowth. Cortical neurons were not treated (no), treated without (mock), or treated with siRNAs specific for SFPQ, NonO, PSPC1, WDR5, NDUFV2, SYT1, Hsc70, and HistH1e. Neurons were then treated without or with antibody 557. (**a**) Mean values + SEM from three independent experiments are shown for total neurite lengths (**** *p* < 0.0001 relative to L1 antibody-stimulated mock-transfected neurons, #### *p* < 0.0001 relative to non-stimulated mock-transfected neurons; one-way ANOVA with Dunn´s multiple comparison test). (**b**) Western blot analysis of lysates from mock-transfected neurons or neurons transfected with siRNAs using the corresponding antibodies. Ponceau S staining of a prominent 35-kDa band served as loading control. (**c**) Immunostaining of mock-transfected neurons or neurons transfected with NDUFV2 siRNA using a NDUFV2 antibody. Scale bar: 10 µm.

To analyze whether the L1 binding partners HistH1e, NDUFV2, SFPQ, NonO, PSPC1, WDR5, Hsc70, or SYT1, which bind to KDET, are involved in L1-dependent neurite outgrowth, neurite outgrowth was determined after transfection of cortical neurons with siRNA for these KDET-binding proteins. The reduction of HistH1e, NDUFV2, SFPQ, NonO, PSPC1, WDR5, Hsc70, or SYT1 expression had no effect on unstimulated neurons, while reduced HistH1e, NDUFV2, SFPQ, NonO, WDR5, Hsc70, and SYT1 expression, but not reduced PSPC1 expression, inhibited the L1 antibody-promoted neurite outgrowth (Figure 5a). Western blot analysis of cell lysates after siRNA treatment showed that the siRNAs reduced levels of HistH1e (26 ± 6% of control), SFPQ (20 ± 19% of control), NonO (36 ± 8% of control), PSPC1 (37 ± 3% of control), WDR5 (30 ± 4% of control), Hsc70 (33 ± 4% of control), and SYT1 (22 ± 3% of control) (Figure 5b). NDUFV2 was not detectable in the lysates. We then determined the NDUFV2 levels by immunofluorescence and observed less fluorescence in siRNA-transfected neurons relative to mock-transfected neurons (Figure 5c). Quantification of the fluorescence showed reduced NDUFV2 levels (36 ± 14%) in siRNA-transfected neurons. These results indicate that the interaction of L1 with HistH1e, NDUFV2, SFPQ, NonO, WDR5, Hsc70, and SYT1 via the KDET motif is required for L1-dependent neurite outgrowth, while the interaction of L1 with PSPC1 is not needed for L1-promoted neurite outgrowth.

## 3. Discussion

In this study, we showed that HistH1e, NDUFV2, WDR5, SFPQ, NonO, PSPC1, Hsc70, and SYT1 interact with L1’s KDET motif. L1’s interactions with these and other proteins, namely TOP1, ERα, RXRβ, PPARγ, VDR, and AR, were shown by ELISA and proximity ligation, which detects L1-interacting proteins at a distance of less than 40. Proximity ligation may lead to the identification of the multiplicity of L1’s binding partners. Yet, the predominant goal of the proximity ligation assay was to show that the molecules of interest should be detected in a cellular context as being close to each other. We did not aim at finding new L1 interaction partners.

The interaction between L1 and HistH1e was reduced by the γ-secretase inhibitor DAPT. Since the generation of L1-55 by γ-secretase and the interactions of L1-55 with its binding partners MeCP2 and HP1 has been shown to be reduced by DAPT [26,27], we conclude that HistH1e interacts with L1-55. Co-immunoprecipitation verified this notion. The interaction of L1 with NDUFV2, WDR5, SFPQ, NonO, PSPC1, Hsc70, and SYT1 was not affected by DAPT, indicating that these KDET-binding proteins do not interact with L1-55, but associate with other known or yet unidentified L1 fragments. Previously [17] and in this study, we showed that NDUFV2 interacts with L1-70. SFPQ and NonO have also been reported to associate with L1-70 [28].

HistH1e interacts with HP1α, HP1β, and HP1γ [33] and competes with MeCP2 for common chromatin binding sites [34]. It is thus conceivable that the interplay between L1-55, MeCP2, HP1, and HistH1e could regulate L1-, MeCP2-, HP1-, and HistH1e-dependent functions during development and in adulthood. L1-55 interacts with HistH1e, MeCP2, and HP1 isoforms via its KDET motif. The interaction between L1 and HistH1e is affected by the tat-KDET peptide in non-stimulated neurons, as seen for the interaction of L1 with MeCP2 [27], while the interaction of L1 with NDUFV2, WDR5, SFPQ, NonO, PSPC1, Hsc70, or SYT1 is affected by the tat-KDET peptide in stimulated neurons, as seen for the L1/HP1 interactions [27]. It is conceivable that the tat-KDET peptide could not bind to HP1, NDUFV2, WDR5, SFPQ, NonO, PSPC1, Hsc70, or SYT1 under basal conditions and thus could not compete with the binding of L1 to these binding partners in non-stimulated neurons. Only after stimulation, the peptide binds to HP1, NDUFV2, WDR5, SFPQ, NonO, PSPC1, Hsc70, or SYT1 and interfere with the binding of L1 to these proteins. However, it is noteworthy that the peptide binds to MeCP2 and HistH1e in non-stimulated neurons to reduce the binding of MeCP2 and HistH1e to L1.

KDET is present in the L1-ICD sequences of mammalian, amphibian, avian, reptilian, and fish species, suggesting that this motif, being conserved in evolution, plays a crucial role in regulating L1 functions. Here, we show that the KDET motif is essential for the regulation of L1-dependent neural functions, such as neurite outgrowth and neuronal survival. Disturbance of the interaction between L1 and its binding partners via the KDET motif prevents neurite outgrowth and neuronal protection against oxidative stress. Moreover, reduction of NDUFV2, WDR5, SFPQ, NonO, Hsc70, and SYT1 expression, and thus reduction of the level of the interactions between L1 and these KDET-binding proteins, inhibits L1-dependent neurite outgrowth. Of note, the reduction of PSPC1 expression does not affect L1-dependent neurite outgrowth, indicating that KDET-mediated interaction of L1 with PSPC1 is not required for L1-dependent neurite outgrowth.

SYT1, which functions as calcium sensors in vesicular trafficking and exocytosis of neurotransmitters and hormones, is involved in the regulation of neurite outgrowth [35,36,37] and it is linked to a rare neurodevelopmental disorder known as SYT1-associated neurodevelopmental disorder or Baker-Gordon syndrome [38]. Besides other symptoms, patients show intellectual disability, poor speech, and delayed development of walking. Similar symptoms are observed in patients with L1 syndrome.

In utero suppression of NDUFV2 expression inhibits migration of cortical neurons and impairs the actin and tubulin cytoskeleton in cortical neurons [39]. NDUFV2 is associated with schizophrenia, bipolar disorder, and Parkinson’s disease (for references, see [39]).

WDR5 plays a crucial role in regulating neuronal gene expression and neurite outgrowth and contributes to the development of X chromosome-linked mental retardation [40].

Mutations in HistH1e cause a rare neurodevelopmental disorder known as Rahman syndrome, which is characterized in addition to other symptoms by intellectual disability and autism spectrum disorder [41].

SFPQ is a key regulator of long neuronal gene expression and thus plays an important role in the pathogenesis of amyotrophic lateral sclerosis, frontotemporal lobar degeneration, and autism spectrum disorder, which are often caused by a dysregulation of the expression of long genes [42]. In addition, SFPQ regulates the expression of mRNAs essential for axon viability and is required for the axonal trafficking of these mRNAs [43].

NonO, which forms heterodimers with SFPQ, is associated with an intellectual disability syndrome, including macrocephaly and a thickened corpus callosum [44].

A Hsc70 inhibitor and Hsc70 siRNA have been reported to decrease neurite outgrowth induced by a neuritogenic reagent [45].

**Figure 6:**
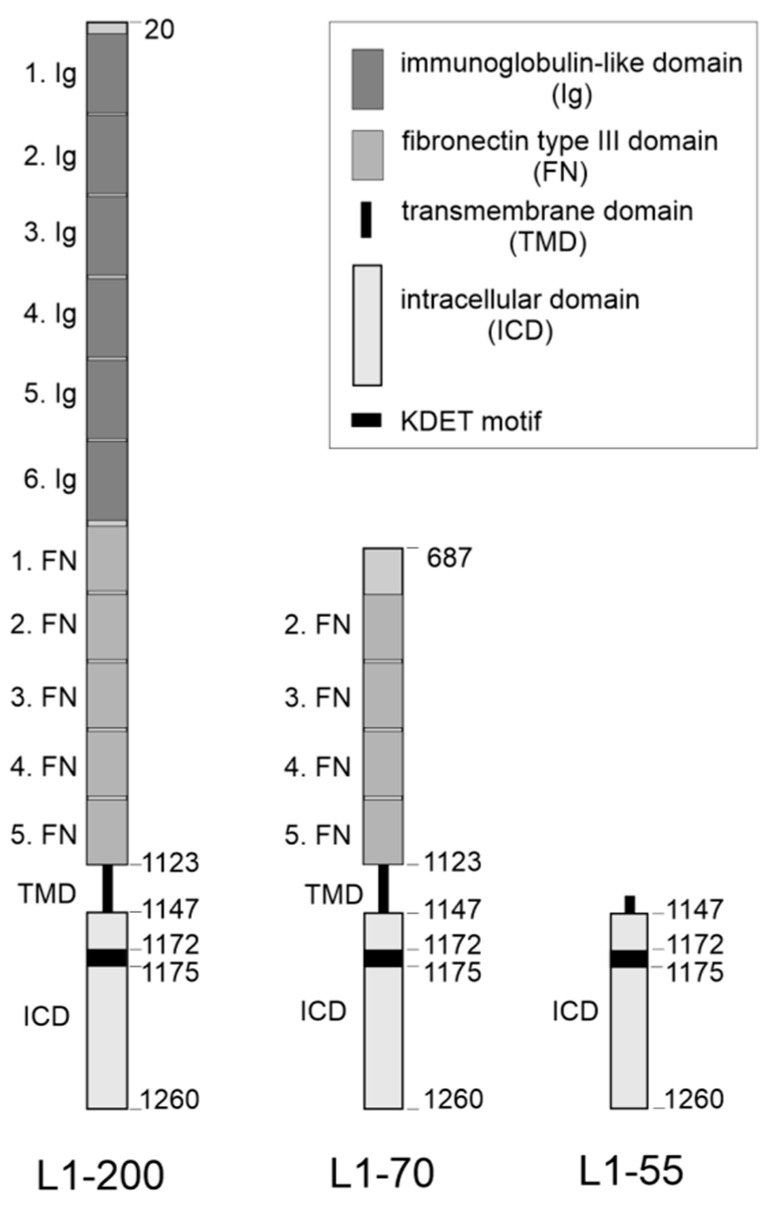
Schematic representation of L1-55 and L1-70. Full-length L1 (L1-200) consists of an extracellular portion with 6 Ig-like (Ig) and 5 FNIII (FN) domains, a transmembrane domain (TMD), and an intracellular domain (ICD), which contains the KDET motif. L1-200 is cleaved by a proteolytically active myelin basic protein in the first FNIII domain at position 687, leading to the generation of L1-70, which comprises part of the first FNIII domain, the second, third, fourth, and fifth FNIII domains, as well as the TMD and ICD with the KDET motif. Sequential cleavage of L1-200 by metalloproteases, the β-site of an amyloid precursor protein cleaving enzyme and ɣ-secretase generates L1-55, which comprises amino acids of the TMD and the entire ICD with the KDET motif.

Based on these findings, we propose that the concomitant or consecutive interactions of L1 with its binding partners via the KDET motif are not only required for regulating L1-dependent cellular functions, such as neurite outgrowth, but also for regulating nervous system development and functions, such as synaptic plasticity, learning and memory, as well as behavior. It is noteworthy, in this context, that the identified L1 binding partners do not display obvious similarities in function but appear to mostly subserve different functional properties. Our study thus expands the spectrum of L1 activities. It is also noteworthy that different fragments of L1, as exemplified by L1-55 and L1-70 (Figure 6), interact with different binding partners via the KDET motif. An explanation for these findings could be that the accessibility of the different binding partners to different fragments is distinct in different compartments and under different metabolic conditions. We propose that other factors determine the binding of different fragments to different molecules. Finally, it is conceivable that the disturbance of the interaction of L1 with its binding partners via the KDET motif contributes to the pathogenesis of L1-associated diseases.

## 4. Materials and Methods

### 4.1. Animals

Mice were bred and maintained at the Universitätsklinikum Hamburg-Eppendorf at 25 °C on a 12 h light/12 h dark cycle with ad libitum access to food and water. C57BL/6J males and females or L1-deficient males [10] and wild-type male littermates were used for all experiments. All animal experiments were conducted in accordance with the German and European Community laws on the protection of experimental animals and approved by the local authorities of the State of Hamburg (animal permit numbers ORG 1022). The manuscript was prepared following the ARRIVE guidelines for animal research [46].

### 4.2. Reagents and Antibodies 

The following antibodies were from Santa Cruz Biotechnology (Dallas, TX, USA): mouse L1 antibody C-2 (NCAM-L1; sc-514360; no RRID available) against the intracellular L1 domain, mouse pan hnRNP A antibody C-6 (sc-166577; RRID:AB_2117484), mouse HistH1 antibody AE-4 (sc-8030; RRID:AB_675641), mouse NDUFV2 antibody F-5 (sc-271620; RRID:AB_10707652), goat CHL1 antibody C-18 (sc-34986, RRID:AB_1121563), mouse importin β1 (Karyopherin β1) antibody H-7 (sc-137016; RRID:AB_2133993), goat TOP1 (Topo I) antibody C-15 (sc-5342; RRID:AB_2205741), goat SYT (Synaptotagmin 1) antibody N-19 (sc-7753; RRID:AB_661534), SFPQ (PSF) antibody H80 (sc-28730; RRID:AB_2186937), rabbit WDR5 antibody H-36 (sc-135245; RRID:AB_10708710), rabbit hnRNP A isoform (pan hnRNP A) antibody H-200 (sc-15385; RRID:AB_648311), rabbit Nup93 antibody H-300 (sc-292099; RRID:AB_10844043), rabbit AR (androgen receptor) antibody N-20 (sc-816; RRID:AB_1563391), rabbit VDR (vitamin D receptor) antibody C-20 (sc-1008; RRID:AB_632070), rabbit RXR (retinoid X receptors) antibody C-20 (sc-831; RRID:AB_632375), and rabbit ER (estrogen receptor) α antibody HC-20 (sc-543; RRID:AB_631471). Rabbit antibodies against PSPC1 (16714-1-AP; Proteintech Cat# 16714-1-AP, RRID:AB_2878302), NonO (11058-1-AP; Proteintech Cat# 11058-1-AP, RRID:AB_2152167), NDUFV2 (15301-1-AP; RRID:AB_2149048), Hsc70 (10654-1-AP; RRID:AB_2120153) and Histone H1 (18201-1-AP; RRID:AB_10859820) were from ChromoTek & Proteintech Germany (Planegg-Martinsried, Germany). Rabbit antibodies against PPARγ (#2492; RRID:AB_2335662), MeCP2 (D4F3; #3456, RRID:AB_2143849), and HP1ɣ (#2619, RRID:AB_2070984) were from Cell Signaling Technology Europe (Leiden, The Netherlands). Rat monoclonal function-stimulating L1 antibody 557 has been described [31]. The anti-L1CAM antibody ab12399 against the intracellular L1 domain was from Abcam (Berlin, Germany). Secondary antibodies were from Dianova (Hamburg, Germany).

Production and purification of the recombinant His-tagged intracellular domain of mouse L1 (L1CAM_MOUSE, P11627; aa 1147-1260) (L1-ICD) and of the mouse close homolog of L1 (CHL1-ICD) have been described [47,48,49].

Recombinant His-tagged HP1α (human CBX5; Cay-11235) was from Biomol (Hamburg, Germany). Recombinant human SFPQ (amino acids 1-300; SFPQ-2617H) with His-tag, recombinant human NonO (amino acids 1-471; NONO-1332H) with His/sumo-tag, recombinant human PRPC1 (amino acids 1-351; PSPC1-2038H) with His-tag, recombinant full-length human AR (AR-991H) with GST-tag, recombinant human ERα (amino acids 65-280; ESR1-12557H) with GST-tag, recombinant human VDR (amino acids 128-427; VDR-3659H) with His-tag, recombinant human WD Repeat Domain 5 with His-tag (amino acids 1-334; WDR5-2734H), and recombinant human PPARγ (amino acids 209-477; PPARG-2772H) with His-tag were from BioMart (Shirley, NY, USA). Recombinant full-length human NDUFV2 (AR51137PU-S; amino acids 33-249 fused to a His-tag) was from OriGene Technologies (Rockville, MD, USA), recombinant human DNA Topoisomerase-I (70 kDa, Sf9 Insect Cells; MBS145206) was from Biozol (Eching, Germany), and recombinant full-length human RXRβ (BML-SE127-0050) was from Enzo Life Sciences (Lörrach, Germany). Purified bovine histone H1 (14-155) was from Merck (Darmstadt, Germany). Recombinant human HSC70 with His-tag (SMQ-SPR-106A), human synaptotagmin 1 containing amino acids 136-382 (ABX263211) and full-length human PPARγ (PPARgamma FL; Cay61700-25) were from Biozol.

Synthetic KDET peptide (DSEARPMKDETFGE), QNQS peptide (DSEARPMQNQSFGE), tat-KDET peptide (YGRKKRRQRRRDSEARPMKDETFGE), which comprises the cell-penetrating sequence YGRKKRRQRRR [50] from the transactivator of transcription (tat) protein of the human immunodeficiency virus and the L1-ICD sequence DSEARPMKDETFGE, and tat-QNQS peptide (YGRKKRRQRRRDSEARPMQNQSFGE), which comprises the tat sequence and the mutated L1-ICD sequence DSEARPMQNQSFGE, were from Schafer-N (Copenhagen, Denmark). DAPT (Cay13197-5; CAS 208255-80-5) was from Biomol (Hamburg, Germany). DAPI was from Thermo Fisher Scientific (Darmstadt, Germany). The siRNAs for murine WDR5 (sc-61799), SFPQ (PSF; sc-38305), NonO (p54/nrb; sc-38164), PSPC1 (sc-152566), PPARγ (PPARgamma; sc-29456), HistHe (Histone cluster 1 H1E; sc-37975), NDUFV2 (sc-149892), Hsc70 (HSC 70; sc-35593), and SYT1 (Synaptotagmin I; sc-41311) were from Santa Cruz Biotechnology.

### 4.3. ELISA

For ELISA, 25 µL of 10 or 20  µg/mL recombinant proteins were incubated overnight at 4 °C in 384-well microtiter plates with a high-binding surface (Corning, Tewksbury, MA, USA). All of the following steps were performed at room temperature. Wells were washed with Dulbecco´s phosphate-buffered saline with MgCl_2_ and CaCl_2_ (Sigma-Aldrich, Taufkirchen, Germany) (PBS), treated with blocking solution (2% essentially fatty acid-free bovine serum albumin in PBS) for 2  h, washed again with PBS containing 0.005% Tween 20 (PBST), and incubated with increasing concentrations of recombinant His-tagged L1-ICD or CHL1-ICD as ligands for 1 h under gentle agitation. For competition ELISA, 2.5 µM L1-ICD was preincubated for 1 h without or with a 5-fold molar excess of L1 peptides KDET or QNQS. The mixtures were then incubated with substrate-coated recombinant proteins. After washing two times with PBS and three times with PBST, L1 antibody C-2 (1:500) or CHL1 antibody C-18 (1:200) in blocking solution were applied for 1 h, followed by two washes with PBS and three washes with PBST, and incubation with horseradish peroxidase-coupled anti-mouse antibody (diluted 1:2000 in blocking solution) for 1 h. Wells were washed again with PBST, and 1 mg/mL ortho-phenylenediamine dihydrochloride (Thermo Fisher Scientific) was used for the detection of bound L1-ICD or CHL1-ICD. The reaction was terminated by the addition of 25 µL of 2.5 M sulfuric acid. Absorbance was measured at 492  nm with an ELISA reader (µQuant; BioTek, Bad Friedrichshall, Germany).

### 4.4. Cultures of Cerebellar and Cortical Neurons

Cerebellar neurons were prepared from the cerebella of 6- to 8-day-old mice. Cerebella were incubated with 10 mg/mL trypsin and 0.5 mg/mL DNase I (Sigma-Aldrich) in Hanks’ balanced salt solution (HBSS) for 15 min at 37 °C, washed with HBSS, mechanically dissociated, and centrifuged at 100× *g* for 15 min. Cells were then diluted in Neurobasal A medium (Thermo Fisher Scientific), supplemented with 2 mM L-glutamine (Thermo Fisher Scientific), 4 nM L-thyroxine (Sigma-Aldrich), 0.1 mg/mL BSA (Sigma-Aldrich), 12.5 μg/mL insulin (Sigma-Aldrich), 30 nM sodium selenite (Sigma-Aldrich), 100 μg/mL transferrin, 0.1 mg/mL streptomycin, and 100 U/mL penicillin (Thermo Fisher Scientific). For the proximity ligation assay, cells were seeded onto poly-L-lysine-coated 12 mm coverslips in a 24-well plate at a density of 2.5 × 10^5^ cells per well. For the determination of neurite outgrowth, cells were seeded at a density of 5 × 10^4^ cells per well of a 48-well plate coated with poly-L-lysine (Sigma-Aldrich). For neuronal survival analysis, cells were seeded at a density of 1.25 × 10^5^ cells per well of a 48-well plate coated with poly-L-lysine.

For the culturing of cortical neurons, cerebral cortices were dissected from 15.5- to 16.5-day-old embryos and incubated in 0.025% trypsin (Sigma-Aldrich) in HBSS at 37 °C for 30 min. The cortices were then incubated in HBSS containing 1% BSA (Sigma-Aldrich) and 1% trypsin inhibitor (T-6522, Sigma-Aldrich) at 37 °C for 5 min. After washing in HBSS, the tissue was mechanically dissociated, and the dissociated cells were cultured in Neurobasal medium (Thermo Fisher Scientific) supplemented with 1% B-27 (Thermo Fisher Scientific), 2 mM L-glutamine (Thermo Fisher Scientific), 100 U/mL penicillin (Thermo Fisher Scientific), and 100 μg/mL streptomycin (Thermo Fisher Scientific). For the proximity ligation assay and immunostaining, cells were seeded onto poly-L-lysine-coated 12 mm coverslips in a 24-well plate at a density of 2.5 × 10^5^ cells per well. For the determination of neurite outgrowth, cells were seeded at a density of 5 × 10^4^ cells or 1 × 10^6^ per well of a 48-well plate coated with poly-L-lysine (Sigma-Aldrich). For Western blot analysis, cells were seeded onto poly-L-lysine-coated 12-well plates at a density of 1.5 × 10^6^ cells per well.

To stimulate L1 functions, neurons were treated with 50 µg/mL L1 antibody 557. For DAPT treatment, neurons were treated for 2 h after seeding with 10 µM DAPT in DMSO (final concentration: 0.1%). Treatment of neurons with 50 µg/mL tat-KDET or tat-QNQS peptide was conducted 30 min after seeding.

For transfection of cortical neurons, cortical neurons were seeded onto poly-L-lysine-coated coverslips in 24-well plates (proximity ligation and immunostaining) or onto 12-well plates (Western blot analysis) and 48-well plates (neurite outgrowth) and maintained for 2 h before transfection with 1 µL (48-well), 2 µL (24-well), or 4 µL (12-well) of 10 µM siRNA and 1-4 µL FuGENE transfection reagent per well. L1 antibody 557 (50 µg/mL) was added to the cultures 24 h after transfection (for proximity ligation and cell survival) or 2 h after transfection (neurite outgrowth), and 48 h after transfection, the cells were analyzed for neurite outgrowth and cell survival, or used for immunostaining, Western blot analysis, or proximity ligation assay.

### 4.5. Proximity Ligation Assay and Immunostaining with Cerebellar and Cortical Neurons

Cultures were fixed for 15 min at room temperature in 4% formaldehyde, washed with PBS, and used for immunostaining or subjected to a proximity ligation assay using Duolink PLA products according to the manufacturer’s protocol (Sigma-Aldrich; Duolink PLA technology) with minor modifications. For the proximity ligation assay, cells were incubated with 1% Triton X-100 in PBS for 30 min, washed once with PBS, blocked with Duolink Blocking solution for 30 min, and incubated for 24 h at 4 °C with mouse L1 antibody C-2 and goat or rabbit antibodies against TOP1, SYT, SFPQ, WDR5, hnRNP A isoforms, Nup93, PSPC1, NonO, NDUFV2, HistH1e, ERα, RXR, PPARγ, AR, or VDR, or with rabbit L1 antibody 12399 and mouse antibodies against impβ1 or hnRNP A, all diluted 1:10 in Duolink Antibody Diluent. Cells were washed two times using Duolink Wash Buffer A and incubated with a mixture of secondary antibodies conjugated with oligonucleotides (Duolink PLA Anti-Rabbit or Anti-Goat Probe MINUS and Duolink Anti-Mouse PLA Probe PLUS). The proximity ligation reaction was then performed according to the manufacturer’s protocol using the Duolink In Situ Detection Reagent RED. Thereafter, the coverslips were incubated with 5 µg DAPI/mL in PBS for 15 min, washed twice with PBS, and mounted in Immuno-Mount (Thermo Fisher Scientific). Ten images per condition were taken using an Olympus F1000 confocal microscope and analyzed using ImageJ software (ImageJ version 1.53q; https://imagej.nih.gov/ij/index.html; RRID:SCR_003070, 30 March 2022). Numbers of red dots and numbers of DAPI-stained nuclei were determined using ImageJ, and the number of dots per image was divided by the number of nuclei per image to determine the average number of dots per nucleus. The average values were determined from 10 images per condition.

For immunostaining, fixed cells were incubated with 1% Triton X-100 in PBS for 30 min, washed once with PBS, blocked with Duolink Blocking solution for 30 min, and incubated for 24 h at 4 °C with mouse NDUFV2 antibody diluted 1:10 in Duolink Antibody Diluent. Cells were then washed three times using PBS and incubated with an anti-mouse Cy3-conjugated secondary antibody.

### 4.6. Determination of Neurite Outgrowth and Neuronal Survival

Cells were washed gently with pre-warmed culture medium, fixed in 2.5% glutaraldehyde for 1 h at room temperature, and stained with 1% toluidine blue and 1% methylene blue in 1% sodium tetraborate for 1 h at room temperature. Neurite outgrowth was analyzed by measuring the total length of neurites in an Axiovert microscope with the AxioVision 4.6 imaging system (Carl Zeiss, Oberkochen, Germany).

To determine cell death, neurons were maintained overnight in serum-free medium and then treated with 50 µg/mL L1 antibody 557 and exposed to oxidative stress by the addition of 10 µM H_2_O_2_ for 24 h. Live and dead cells were then stained with calcein-AM (ThermoFisher Scientific) and propidium iodide (Sigma-Aldrich) and imaged with a Zeiss AxioObserver.A1 microscope (Carl Zeiss) with a 20× objective (aperture 0.4) and the AxioVision 4.6 software (Carl Zeiss). Live and dead cells were counted in five images (containing 350–400 cells each) from each of three wells per condition and experiment using ImageJ (version 1.53q; https://imagej.nih.gov/ij/index.html; RRID:SCR_003070, 30 March 2022).

### 4.7. Immunorecipitation and Western Blot Analysis

For the preparation of nuclear and non-nuclear fractions, the Subcellular Protein Fractionation Kit for Tissue (Thermo Fisher Scientific) was used. Fractions in cytoplasmic extraction buffer (CEB) and membrane extraction buffer (CEB) were pooled and taken as a non-nuclear fraction. Fractions in the nuclear extraction buffer were taken as nuclear fraction. Nuclear and non-nuclear fractions were used for immunoprecipitation.

For immunoprecipitation, Protein G magnetic beads (25 μL per sample) (Dynabeads^TM^ Protein G, Thermo Fisher Scientific) were washed twice in phosphate-buffered saline (PBS), pH 7.4, incubated in a dilution buffer (1 mg/mL bovine serum albumin in PBS) for 10 min at 4 °C under rotation. The mouse HistH1 or NDUFV2 antibodies (10 μg per sample) were diluted in dilution buffer. The diluted antibody solutions were incubated with the beads for 1 h at 4 °C under rotation. The beads were washed in dilution buffer for 5 min at 4 °C under rotation. A freshly prepared 13 mg/mL stock solution of dimethyl pimelimidate (DMP) was 1:1 diluted with wash buffer (0.2 M triethanolamine in PBS). After washing the beads in PBS, the diluted DMP solution (pH 8–9) was added to the beads and incubated for 30 min at room temperature (20–24 °C) under rotation. The beads were washed in a wash buffer for 5 min at room temperature under rotation. The beads were then incubated in DMP solution and wash buffer for two further times. Finally, the beads were incubated twice in quenching buffer (50 mM ethanolamine in PBS) for 5 min at room temperature under rotation. After washing, the beads were incubated with brain fractions overnight at 4°C under rotation. The beads were washed twice with lysis buffer and once with PBS. The beads were then boiled for 5 min in the sample buffer (60 mM Tris-HCl, pH 6.8, 2% SDS, 1% β-mercaptoethanol, 6% glycerol, and 0.01% bromophenol blue).

For Western blot analysis, samples were run on 4–20% Mini-PROTEAN^®^ TGX^™^ Precast Protein Gels (BioRad, Feldkirchen, Germany). PageRuler™ Plus Prestained Protein Ladder (Thermo Fisher Scientific) was used as a molecular weight marker. Proteins were then transferred to 0.45 μm Protran™ nitrocellulose membranes (VWR, Darmstadt, Germany) and stained with Ponceau S (Thermo Fisher Scientific) to control protein loading. The membranes were then incubated for 1 h in blocking solution (5% non-fat dry milk powder in Tris-buffered saline (TBS) (TBS; 10 mM Tris-HCl, pH 7.4; 150 mM NaCl) with 0.05% Tween 20 (TBST)) and then incubated overnight with primary antibodies in blocking solution at 4 °C with shaking. After washing five times for 5 min in TBST, the membranes were incubated for 1 h with horseradish peroxidase-conjugated secondary antibodies (1:20,000 in blocking solution). Bands were detected using enhanced chemiluminescent solution (ECL Prime and Select Western blotting reagents; GE Healthcare, Solingen, Germany) and a CCD camera (ImageQuant LAS-4000 mini; GE Healthcare).

### 4.8. Statistical Analysis

Analyses were performed using SigmaPlot 14.0. The types of tests are indicated in the legends. *p*-Values of <0.05, <0.01, <0.001 and <0.0001 were accepted as a significant difference and indicated by *, **, ***, and **** or #, ##, ###, and #### or §, §§, §§§, and §§§§.

## Figures and Tables

**Table 1:** L1 binding partners assayed in this study.

AR	androgen receptor
ERα	estrogen receptor α
ERβ	estrogen receptor β
HistH1e	histone H1.4
hnRNP A1	heterogeneous nuclear ribonucleoprotein A1
hnRNP A2/B1	heterogeneous nuclear ribonucleoprotein A2/B1
hnRNP A3	heterogeneous nuclear ribonucleoprotein A3
impβ1	importin β1
MeCP2	methyl CpG binding protein 2
NDUFV2	NADH dehydrogenase ubiquinone flavoprotein 2
NonO	non-POU domain containing octamer-binding protein
Nup93	nucleoporin 93 kDa
PPARγ	peroxisome proliferator-activated receptor γ
PSPC1	paraspeckle component 1
RXRβ	retinoid X receptor β
SFPQ	splicing factor proline/glutamine-rich
SYT1	synaptotagmin 1
TOP1	DNA topoisomerase I
VDR	vitamin D receptor
WDR5	WD-repeat protein 5

## Data Availability

Not applicable.

## References

[1] Loers G., Schachner M. (2007). Recognition molecules and neural repair. J. Neurochem..

[2] Maness P.F., Schachner M. (2007). Neural recognition molecules of the immunoglobulin superfamily: Signaling transducers of axon guidance and neuronal migration. Nat. Neurosci..

[3] Schafer M.K., Altevogt P. (2010). L1CAM malfunction in the nervous system and human carcinomas. Cell Mol. Life Sci..

[4] Sytnyk V., Leshchyns’ka I., Schachner M. (2017). Neural cell adhesion molecules of the immunoglobulin superfamily regulate synapse formation, maintenance, and function. Trends Neurosci..

[5] Lutz D., Kataria H., Kleene R., Loers G., Chaudhary H., Guseva D., Wu B., Jakovcevski I., Schachner M. (2016). Myelin basic protein cleaves cell adhesion molecule L1 and improves regeneration after injury. Mol. Neurobiol..

[6] Fransen E., Lemmon V., Van Camp G., Vits L., Coucke P., Willems P.J. (1995). CRASH syndrome: Clinical spectrum of corpus callosum hypoplasia, retardation, adducted thumbs, spastic paraparesis and hydrocephalus due to mutations in one single gene, L1. Eur. J. Hum. Genet..

[7] Fransen E., Vits L., Van Camp G., Willems P.J. (1996). The clinical spectrum of mutations in L1, a neuronal cell adhesion molecule. Am. J. Med. Genet..

[8] Hortsch M., Nagaraj K., Mualla R. (2014). The L1 family of cell adhesion molecules: A sickening number of mutations and protein functions. Adv. Neurobiol..

[9] Zhang L. (2010). CRASH syndrome: Does it teach us about neurotrophic functions of cell adhesion molecules?. Neuroscientist.

[10] Dahme M., Bartsch U., Martini R., Anliker B., Schachner M., Mantei N. (1997). Disruption of the mouse L1 gene leads to malformations of the nervous system. Nat. Genet..

[11] Fransen E., D’Hooge R., Van Camp G., Verhoye M., Sijbers J., Reyniers E., Soriano P., Kamiguchi H., Willemsen R., Koekkoek S.K. (1998). L1 knockout mice show dilated ventricles, vermis hypoplasia and impaired exploration patterns. Hum. Mol. Genet..

[12] Katidou M., Vidaki M., Strigini M., Karagogeos D. (2008). The immunoglobulin superfamily of neuronal cell adhesion molecules: Lessons from animal models and correlation with human disease. Biotechnol. J..

[13] Wei C.H., Ryu S.E. (2012). Homophilic interaction of the L1 family of cell adhesion molecules. Exp. Mol. Med..

[14] Kleene R., Lutz D., Loers G., Bork U., Borgmeyer U., Hermans-Borgmeyer I., Schachner M. (2021). Revisiting the proteolytic processing of cell adhesion molecule L1. J. Neurochem..

[15] Appel F., Holm J., Conscience J.F., Schachner M. (1993). Several extracellular domains of the neural cell adhesion molecule L1 are involved in neurite outgrowth and cell body adhesion. J. Neurosci..

[16] Holm J., Appel F., Schachner M. (1995). Several extracellular domains of the neural cell adhesion molecule L1 are involved in homophilic interactions. J. Neurosci. Res..

[17] Kraus K., Kleene R., Braren I., Loers G., Lutz D., Schachner M. (2018). A fragment of adhesion molecule L1 is imported into mitochondria, and regulates mitochondrial metabolism and trafficking. J. Cell Sci..

[18] Kraus K., Kleene R., Henis M., Braren I., Kataria H., Sharaf A., Loers G., Schachner M., Lutz D. (2018). A fragment of adhesion molecule L1 binds to nuclear receptors to regulate synaptic plasticity and motor coordination. Mol. Neurobiol..

[19] Lutz D., Loers G., Kleene R., Oezen I., Kataria H., Katagihallimath N., Braren I., Harauz G., Schachner M. (2014). Myelin basic protein cleaves cell adhesion molecule L1 and promotes neuritogenesis and cell survival. J. Biol. Chem..

[20] Lutz D., Sharaf A., Drexler D., Kataria H., Wolters-Eisfeld G., Brunne B., Kleene R., Loers G., Frotscher M., Schachner M. (2017). Proteolytic cleavage of transmembrane cell adhesion molecule L1 by extracellular matrix molecule Reelin is important for mouse brain development. Sci. Rep..

[21] Lutz D., Wolters-Eisfeld G., Joshi G., Djogo N., Jakovcevski I., Schachner M., Kleene R. (2012). Generation and nuclear translocation of sumoylated transmembrane fragment of cell adhesion molecule L1. J. Biol. Chem..

[22] Lutz D., Wolters-Eisfeld G., Schachner M., Kleene R. (2014). Cathepsin E generates a sumoylated intracellular fragment of the cell adhesion molecule L1 to promote neuronal and Schwann cell migration as well as myelination. J. Neurochem..

[23] Kalus I., Schnegelsberg B., Seidah N.G., Kleene R., Schachner M. (2003). The proprotein convertase PC5A and a metalloprotease are involved in the proteolytic processing of the neural adhesion molecule L1. J. Biol. Chem..

[24] Mechtersheimer S., Gutwein P., Agmon-Levin N., Stoeck A., Oleszewski M., Riedle S., Postina R., Fahrenholz F., Fogel M., Lemmon V. (2001). Ectodomain shedding of L1 adhesion molecule promotes cell migration by autocrine binding to integrins. J. Cell Biol..

[25] Congiu L., Granato V., Loers G., Kleene R., Schachner M. (2022). Mitochondrial and neuronal dysfunctions in L1 mutant mice. Int. J. Mol. Sci..

[26] Kleene R., Loers G., Castillo G., Schachner M. (2022). Cell adhesion molecule L1 interacts with the chromo shadow domain of heterochromatin protein 1 isoforms alpha, beta, and via its intracellular domain. FASEB J..

[27] Loers G., Kleene R., Girbes Minguez M., Schachner M. (2022). The cell adhesion molecule L1 interacts with methyl CpG binding protein 2 via its intracellular domain. Int. J. Mol. Sci..

[28] Girbes Minguez M., Wolters-Eisfeld G., Lutz D., Buck F., Schachner M., Kleene R. (2020). The cell adhesion molecule L1 interacts with nuclear proteins via its intracellular domain. FASEB J..

[29] Hu J., Lin S.L., Schachner M. (2022). A fragment of cell adhesion molecule L1 reduces amyloid-beta plaques in a mouse model of Alzheimer’s disease. Cell Death Dis..

[30] Leshchyns’ka I., Sytnyk V., Richter M., Andreyeva A., Puchkov D., Schachner M. (2006). The adhesion molecule CHL1 regulates uncoating of clathrin-coated synaptic vesicles. Neuron.

[31] Appel F., Holm J., Conscience J.F., von Bohlen und Halbach F., Faissner A., James P., Schachner M. (1995). Identification of the border between fibronectin type III homologous repeats 2 and 3 of the neural cell adhesion molecule L1 as a neurite outgrowth promoting and signal transducing domain. J. Neurobiol..

[32] Kataria H., Lutz D., Chaudhary H., Schachner M., Loers G. (2016). Small molecule agonists of cell adhesion molecule L1 mimic L1 functions in vivo. Mol. Neurobiol..

[33] Daujat S., Zeissler U., Waldmann T., Happel N., Schneider R. (2005). HP1 binds specifically to Lys26-methylated histone H1.4, whereas simultaneous Ser27 phosphorylation blocks HP1 binding. J. Biol. Chem..

[34] Ghosh R.P., Horowitz-Scherer R.A., Nikitina T., Shlyakhtenko L.S., Woodcock C.L. (2010). MeCP2 binds cooperatively to its substrate and competes with histone H1 for chromatin binding sites. Mol. Cell Biol..

[35] Fukuda M., Mikoshiba K. (2000). Expression of synaptotagmin I or II promotes neurite outgrowth in PC12 cells. Neurosci. Lett..

[36] Kabayama H., Takei K., Fukuda M., Ibata K., Mikoshiba K. (1999). Functional involvement of synaptotagmin I/II C2A domain in neurite outgrowth of chick dorsal root ganglion neuron. Neuroscience.

[37] Mikoshiba K., Fukuda M., Ibata K., Kabayama H., Mizutani A. (1999). Role of synaptotagmin, a Ca2+ and inositol polyphosphate binding protein, in neurotransmitter release and neurite outgrowth. Chem. Phys. Lipids.

[38] Baker K., Gordon S.L., Melland H., Bumbak F., Scott D.J., Jiang T.J., Owen D., Turner B.J., Boyd S.G., Rossi M. (2018). SYT1-associated neurodevelopmental disorder: A case series. Brain.

[39] Chen T., Wu Q., Zhang Y., Zhang D. (2015). NDUFV2 regulates neuronal migration in the developing cerebral cortex through modulation of the multipolar-bipolar transition. Brain Res..

[40] Nakagawa T., Xiong Y. (2011). X-linked mental retardation gene CUL4B targets ubiquitylation of H3K4 methyltransferase component WDR5 and regulates neuronal gene expression. Mol. Cell.

[41] Tremblay M.W., Green M.V., Goldstein B.M., Aldridge A.I., Rosenfeld J.A., Streff H., Tan W.D., Craigen W., Bekheirnia N., Al Tala S. (2022). Mutations of the histone linker H1-4 in neurodevelopmental disorders and functional characterization of neurons expressing C-terminus frameshift mutant H1.4. Hum. Mol. Genet..

[42] Takeuchi A., Iida K., Tsubota T., Hosokawa M., Denawa M., Brown J.B., Ninomiya K., Ito M., Kimura H., Abe T. (2018). Loss of Sfpq causes long-gene transcriptopathy in the brain. Cell Rep..

[43] Cosker K.E., Fenstermacher S.J., Pazyra-Murphy M.F., Elliott H.L., Segal R.A. (2016). The RNA-binding protein SFPQ orchestrates an RNA regulon to promote axon viability. Nat. Neurosci..

[44] Mircsof D., Langouet M., Rio M., Moutton S., Siquier-Pernet K., Bole-Feysot C., Cagnard N., Nitschke P., Gaspar L., Znidaric M. (2015). Mutations in NONO lead to syndromic intellectual disability and inhibitory synaptic defects. Nat. Neurosci..

[45] Cheng L., Wang Y., Xiang L., Qi J. (2021). Heat shock cognate 70 kDa protein is the target of tetradecyl 2,3-dihydroxybenzoate for neuritogenic effect in PC12 Cells. Biomedicines.

[46] Kilkenny C., Browne W., Cuthill I.C., Emerson M., Altman D.G. (2010). Animal research: Reporting in vivo experiments: The ARRIVE guidelines. J. Gene Med..

[47] Kleene R., Cassens C., Bahring R., Theis T., Xiao M.F., Dityatev A., Schafer-Nielsen C., Doring F., Wischmeyer E., Schachner M. (2010). Functional consequences of the interactions among the neural cell adhesion molecule NCAM, the receptor tyrosine kinase TrkB, and the inwardly rectifying K+ channel KIR3.3. J. Biol. Chem..

[48] Andreyeva A., Leshchyns’ka I., Knepper M., Betzel C., Redecke L., Sytnyk V., Schachner M. (2010). CHL1 is a selective organizer of the presynaptic machinery chaperoning the SNARE complex. PLoS ONE.

[49] Xiao M.F., Xu J.C., Tereshchenko Y., Novak D., Schachner M., Kleene R. (2009). Neural cell adhesion molecule modulates dopaminergic signaling and behavior by regulating dopamine D2 receptor internalization. J. Neurosci..

[50] Schwarze S.R., Dowdy S.F. (2000). In vivo protein transduction: Intracellular delivery of biologically active proteins, compounds and DNA. Trends Pharmacol. Sci..

